# EV71 3C protease cleaves host anti-viral factor OAS3 and enhances virus replication

**DOI:** 10.1016/j.virs.2022.04.013

**Published:** 2022-05-03

**Authors:** Xiaolei Zhou, Li Tian, Jian Wang, Baisong Zheng, Wenyan Zhang

**Affiliations:** Center for Infectious Diseases and Pathogen Biology, Institute of Virology and AIDS Research, Key Laboratory of Organ Regeneration and Transplantation of the Ministry of Education, The First Hospital of Jilin University, Jilin, 130021, China

**Keywords:** Enteroviruses, EV71, 3C protease, Cleavage, Anti-viral factor, OAS3

## Abstract

The global spread of enteroviruses (EVs) has become more frequent, severe and life-threatening. Intereron (IFN) I has been proved to control EVs by regulating IFN-stimulated genes (ISG) expression. 2′-5′-oligoadenylate synthetases 3 (OAS3) is an important ISG in the OAS/RNase L antiviral system. The relationship between OAS3 and EVs is still unclear. Here, we reveal that OAS3, superior to OAS1 and OAS2, significantly inhibited EV71 replication *in vitro*. However, EV71 utilized autologous 3C protease (3C^pro^) to cleave intracellular OAS3 and enhance viral replication. Rupintrivir, a human rhinovirus 3C protease inhibitor, completely abolished the cleavage of EV71 3C^pro^ on OAS3. And the proteolytically deficient mutants H40G, E71A, and C147G of EV71 3C^pro^ also lost the ability of OAS3 cleavage. Mechanistically, the Q982-G983 motif in C-terminal of OAS3 was identified as a crucial 3C^pro^ cutting site. Further investigation indicated that OAS3 inhibited not only EV71 but also Coxsackievirus B3 (CVB3), Coxsackievirus A16 (CA16), Enterovirus D68 (EVD68), and Coxsackievirus A6 (CA6) subtypes. Notably, unlike other four subtypes, CA16 3C^pro^ could not cleave OAS3. Two key amino acids variation Ile36 and Val86 in CA16 3C^pro^ might result in weak and delayed virus replication of CA16 because of failure of OAS and 3AB cleavage. Our works elucidate the broad anti-EVs function of OAS3, and illuminate a novel mechanism by which EV71 use 3C^pro^ to escape the antiviral effect of OAS3. These findings can be an important entry point for developing novel therapeutic strategies for multiple EVs infection.

## Highlights

• OAS3, superior to OAS1 and OAS2, broadly inhibits enteroviruses replication.
• EV71 utilizes autologous 3C protease to cleave OAS3 and evade innate immune.
• Two amino acids variation of CA16 3C protease impairs OAS and 3AB cleavage.

## 1 Introduction

Enterovirus 71 (EV71), as the main pathogen causing hand-foot-mouth disease (HFMD), belongs to a genus of small, single-stranded, positive-sense RNA viruses of the family *Picornaviridae* (Muehlenbachs et al., 2015; Baggen et al., 2018). There are 13 species of EVs, 7 of which are human pathogens and contain more than 250 subtypes. These enteroviruses include many important pathogens, such as coxsackieviruses, echoviruses, numbered enteroviruses and rhinoviruses. These diverse viruses cause a variety of diseases, including non-specific febrile illness, hand-foot-and-mouth disease, neonatal sepsis-like disease, encephalitis, paralysis and respiratory diseases. In the past decade, several EV subtypes, such as EV71, Coxsackievirus A16 (CA16), Coxsackievirus B3 (CVB3), Enterovirus D68 (EVD68), and Coxsackievirus A6 (CA6), have garnered increasing attention due to serious public health concerns (Solomon et al., 2010; Pollack et al., 2015; Pons-Salort et al., 2015). And as the representative subtype, EV71 has become the subject of intensive clinical and epidemiological research. Structurally, the genome of EV71 is a ∼7.4 ​kb long, single-stranded, positive RNA (Laitinen et al., 2016). The whole genome contains an open reading frame, which encodes a large polypeptide. The polyprotein is further cleaved into P1, P2, and P3 precursor proteins. The P1 precursor protein is proteolyzed into four structural proteins (VP1, VP2, VP3, and VP4). These structural proteins form the viral capsid, which encloses and protects the RNA genome. The P2 and P3 precursor proteins are proteolyzed into seven non-structural proteins (2A–2C and 3A–3D). These nonstructural proteins are actively involved in almost every stage of viral replication (Lin et al., 2009). Among them, EV71 nonstructural protein 3C (3C^pro^) is a protease that primarily processes the EV71 precursor protein for replication. Previous research has demonstrated that EV 3C^pro^ targets the cleavage sites of Gln/Gly, Gln/Ala, or Gln/Ser scissile pairs in general (Kitamura et al., 1981). Recent evidence shows that the 3C^pro^ can cleave cellular CstF-64 protein, which subsequently halts host RNA processing and polyadenylation (Weng et al., 2009). EV71 also regulates IFN production via the cleavage of IRF7 by 3C^pro^ (Lei et al., 2013). In addition, EV71 3C^pro^ also has been proved to inhibit pyroptosis through the cleavage of gasdermin D (Lei et al., 2017). Whether EV71 3C^pro^ has other cleavage targets and how it relates with virus replication remains to be further studied.

IFN-I plays an important role in controlling the replication of EV71 through promoting the expression of IFN-stimulated genes (ISGs) (Lu et al., 2012, 2015). 2′-5′-oligoadenylate synthetases (OAS) is an important family of IFN-I induced ISGs that offer protection against a wide spectrum of RNA and DNA viruses (Silverman, 2007). In humans, the OAS family consists of four members: OAS1, OAS2, OAS3, and OASL (Kristiansen et al., 2011). OAS1, OAS2, and OAS3 possess 2-5A synthetase activity, whereas OASL is absent from this activity despite sharing significant sequence similarity with the other OAS proteins (Ibsen et al., 2015). When binding to viral dsRNA, OAS1, 2, and 3 could synthesize 2′-5′-phosphodiester-linked 2-5As (Justesen et al., 2000; Hovanessian and Justesen, 2007). The 2-5As are second messengers that bind to and activate the latent endoribonuclease RNase L (Zhou et al., 1993). The RNase L activation restricts viral replication by degrading viral and cellular RNA. Although the antiviral role of OAS/RNase L system has been widely documented on numerous viruses *in vitro* and *in vivo*, the role of OAS/RNase L in EVs infection, especial for EV71, CA16, CVB3, EVD68, and CA6 remains to be identified. And the interplay between EVs infection and intracellular OAS remains to be clarified.

In our study, we revealed that OAS3, with the highest 2-5A synthesis activity, exhibited higher anti-EV71 effect than OAS1 and OAS2. The antiviral effect of OAS3 could extend to other EV subtypes, such as CVB3, CA16, EVD68, and CA6. On the other hand, EV71 could utilize autologous 3C^pro^ to cleave intracellular OAS3 and enhance viral replication. The proteolytic activity deficient mutants H40G, E71A, and C147G of EV71 3C^pro^ lost the ability of OAS3 cleavage. The Q982-G983 motif in OAS3 was identified as a crucial 3C^pro^ cutting site. And two key amino acid variations affect the ability of EV71 3C^pro^ to cleave OAS. We presumed that 3C^pro^ variation might result in divergent replication process of EV subtypes.

## 2 Materials and methods

### 2.1 Cells and viruses

Human HEK293T cells (ATCC Catalog No. CRL-11268) and HeLa cells were purchased from the American Type Culture Collection (Manassas, VA, USA) and cultured in Dulbecco's modiﬁed Eagle's medium (DMEM; Gibco, Amarillo, TX, USA) supplemented with 10% fetal bovine serum (FBS; Gibco BRL, Grand Island, NY, USA), 1 ​mmol/L sodium pyruvate, 100 ​μg/mL penicillin, and 100 ​μg/mL streptomycin at 37 ​°C in a 5% CO_2_ incubator. Enteroviruses EV71, CVB3, CA16, EVD68, and CA6 was preserved in our lab (Xu et al., 2020). The viruses were ampliﬁed using RD cells.

### 2.2 Chemicals

Rupintrivir (#PZ0315-5 ​MG), MG132 (#M7449), and Thapsigargin (Thap, #T9033) were purchased from Merck (Darmstadt, Germany). Bafilomycin A1 (Baf-A1, #HY-100558) were purchased from MedChemExpress (Monmouth Junction, NJ, USA). Other general chemicals were purchased from Sigma (St. Louis, MO, USA), Selleck Chemicals (Houston, TX, USA), and Takara (Shiga, Japan), unless otherwise stated.

### 2.3 Plasmid construction

The *OAS1*, *OAS2*, *and OAS3* gene was amplified from the cDNA of HEK293T cells and then inserted into the VR1012 vector with a C-terminal HA or myc tag. Plasmids containing genes encoding EV71 nonstructural proteins 2A (HA tag, V5 tag), 2B (HA tag), 2C (HA tag), 3A (HA tag), 3B (HA tag), 3AB (HA tag), 3C (HA tag, myc tag), 3D (HA tag), HA-tagged CVB3 3C, CA16 3C, EVD68 3C, and CA6 3C were constructed and maintained in our laboratory. EV71 3C mutants H40G, E71A, C147G, V36I, I86V, and V157I were generated using standard site-directed mutagenesis. OAS3-specific shRNA was inserted into the lentiviral vector pLKO.1 (Addgene, Cambridge, MA, USA). The primer used for mutation and knockdown in this study are shown in Supplementary Table S1. Vector VR1012 was used as the control for gene overexpression and pLKO.1 was used as the control for shRNA-mediated *OAS3* silencing.

### 2.4 Construction of stable silencing cell lines

For the preparation of stably silenced cell lines, HEK293T cells were cotransfected with shOAS3-pLKO.1 or pLKO.1 plus RRE, REV, and VSV-G expression vectors using Lipofectamine 2000 (Invitrogen, Carlsbad, CA, USA) according to the manufacturer's protocol. At 48 ​h after transfection, supernatants containing packaged lentivirus were harvested and used to infect HEK293T cells for 48 ​h. Puromycin (3 ​μg/mL for HEK293T, Sigma) was then added to the culture to screen for stable cell lines.

### 2.5 CCID_50_ determination

The median cell culture infective dose (CCID_50_) was determined as previously described (Xu et al., 2020). Briefly, HEK293T cells in suspension were infected with virus, seeded in 96-well plates, and incubated at 37 ​°C for 1 week to observe the cytopathic effect (CPE). CCID_50_ was calculated following the method described by Reed and Muench.

### 2.6 RNA extraction, reverse transcription, and quantitative real-time PCR (RT-qPCR)

RT-qPCR was determined as previously described (Xu et al., 2020). Briefly, total RNA was extracted and reverse-transcribed, then RT-qPCR was performed with the primers listed in Supplementary Table S2. The threshold cycle value of each sample was determined, and the relative mRNA level was normalized to that of glyceraldehyde-3-phosphate dehydrogenase.

### 2.7 SDS-PAGE, Western blot analysis and antibodies

Cell lysate preparation, SDS-PAGE, and Western blot was performed as previously described (Xu et al., 2020). The following antibodies and reagents were used: human OAS3 (#21915-1-AP, Proteintech, Wuhan, China), anti-HA mouse monoclonal antibody (#F1804, Sigma), anti-V5 monoclonal antibody (#R960-25, Invitrogen), β-tubulin (#RM2003, Ray Antibody Biotech, Beijing, China), goat anti-rabbit peroxidase-conjugated IgG antibody (Millipore; AP132P), goat anti-mouse peroxidase-conjugated IgG Antibody (Millipore; AP124P), goat anti-mouse IgG (H+L) highly cross-adsorbed secondary antibody (Jackson ImmunoResearch, West Grove, PA, USA; 115-055-062), and goat anti-rabbit IgG (H+L) highly cross-adsorbed secondary antibody (Jackson ImmunoResearch; 115-055-003). Polyclonal antibodies against EV71, CA16, CVB3, CA6, and EVD68 were obtained from rabbits immunized with EV71, CA16, CA6, EVD68, and mouse immunized with CVB3 in our laboratory.

### 2.8 Immunofluorescence microscopy

HeLa cells (20%–50% confluent) seeded onto coverslips in 6-well plates were transfected as indicated. At 24 ​h post-transfection, the cells were fixed with 2% formaldehyde, permeabilized with 0.25% Triton X-100, blocked in 10% serum, and incubated with mouse anti-myc mAb (Millipore) and mouse anti-HA mAb (Sigma) diluted 1:1000 for 2 ​h. They were then stained with Alexa Fluor 488-conjugated goat anti-mouse IgG and Alexa Fluor 633-conjugated goat anti-mouse IgG (Molecular Probes, Eugene, OR, USA) diluted 1:1000 for 1 ​h. After washing, the coverslips were mounted with a mounting medium (Sigma). Images were acquired on a Zeiss LSM710 confocal microscope and adjusted using ZEN software (Zeiss, Oberkochen, Germany). The thresholded Mander's overlap coefficients (tMOC) were calculated using FIJI. Unless stated otherwise, measurements were based on a minimum of five cells.

### 2.9 Co-immunoprecipitation (Co-IP)

10-cm dishes of HEK293T cells were transfected as indicated. 48 h post transfection, cells were harvested and lysed with buffer containing a protease inhibitor cocktail (Roche, Indianapolis, IN). Cell lysates were incubated with anti-myc antibody and protein G agarose beads (Life Technologies) at 4 ​°C overnight. After washing and elution, the eluted proteins were obtained by centrifugation, followed by Western blot analysis.

### 2.10 Statistical analyses

Statistical analyses were conducted using SPSS software (version 17.0). Data are reported as the mean ​± ​SD from at least two or three independent experiments performed in triplicate. Statistical signiﬁcance was evaluated using the Student's *t*-test. Significant differences are indicated in the figures as follows: ∗*P* ​< ​0.05, ∗∗*P* ​< ​0.01. # indicates no significance.

## 3 Results

### 3.1 OAS3 inhibits the replication of EV71

**Fig. 1:**
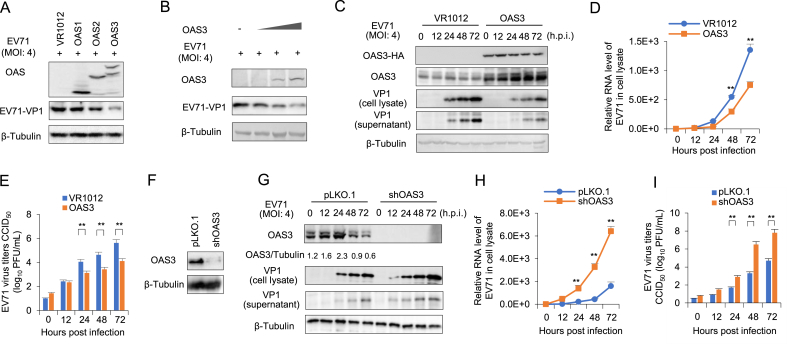
OAS3 suppresses EV71 replication in HEK293T cells. **A** Effect of OAS1, OAS2, OAS3 on EV71 replication. HEK293T cells transfected with OAS1, OAS2, and OAS3 expression plasmids were infected with EV71 at an MOI of 4 for 48 ​h. The expression levels of exogenous OAS and VP1 were examined by Western blot. **B** OAS3 overexpression represses EV71 replication in a dose-dependent manner. HEK293T cells transfected with different doses (100 ​ng, 300 ​ng, 900 ​ng) of OAS3 expression plasmids were infected with EV71 at an MOI of 4 for 48 ​h. The expression levels of exogenous OAS and VP1 were examined by Western blot. **C** HEK293T cells transiently transfected with OAS3 overexpression and control plasmids were infected with EV71 at an MOI of 4. Cells and supernatants were harvested at the indicated time points. The expression levels of exogenous OAS3 and VP1 were examined by Western blot. **D** Viral RNA in cell lysate was examined by RT-qPCR. Cell lysate from HEK293T cells transfected with VR1012 and OAS3 and then infected with EV71 was collected in different time points. **E** Viral titers in the supernatants from the experiment of Fig. 1D were determined by the plaque assay. **F** shRNA-mediated endogenous OAS3 silencing in HEK293T cells. Silencing efficiency was examined by Western blot. **G** OAS3 knockdown enhances EV71 replication. shRNA-mediated OAS3 knockdown (shOAS3) and control HEK293T (pLKO.1) cells were infected with EV71 at an MOI of 4. Cells and supernatants were harvested at the indicated time points. The expression levels of endogenous OAS3 and VP1 were examined by Western blot. **H** Viral RNA in cell lysate from the experiment of Fig. 1G were examined by RT-qPCR. **I** Viral titers in the supernatants from the experiment of Fig. 1G were determined by the plaque assay. The results represent the means ​± ​SD from three independent experiments. Statistical significance was analyzed using Student's *t*-test (∗∗*P* ​< ​0.01).

OAS constitutes an important family of ISGs and virus-induced antiviral restriction factors that offer protection against a wide spectrum of RNA and DNA viruses (Kristiansen et al., 2011). However, the correlation between OAS family members OAS1, OAS2, OAS3, who possess 2-5A synthetic activity, and enterovirus replication is still unknown. Here, HEK293T cells were transfected with OAS expressing plasmids and then infected with EV71. Western blot analysis was performed to test the expression of exogenous OAS and VP1. We found that OAS3 suppressed EV71 replication more strongly than OAS1 and OAS2 (Fig. 1A). And OAS3 inhibited EV71 replication in a dose-dependent manner (Fig. 1B). Also, OAS3 overexpression can continuously reduce EV71 replication in HEK293T cells. The production of viral proteins (presented by VP1), viral RNA and extracellular virions were significantly lower in OAS3-overexpressed cells than in the control cells transfected with VR1012 vector (Fig. 1C–E). For a comprehensive investigation on the role of OAS3 in suppressing EV71 replication, endogenous OAS3 was silenced by shRNA-mediated knockdown in HEK293T cells (Fig. 1F). Then, HEK293T shRNA control (pLKO.1) and shOAS3 cells were infected with EV71 at an MOI of 4 for 0–72 ​h. Western blot and RT-qPCR analysis indicated that OAS3 silencing enhanced intracellular viral RNA transcription and protein expression (Fig. 1G and H). Extracellular viral titers were also upregulated following OAS3 silencing (Fig. 1I). In addition, RNase L activation was examined by agarose electrophoresis of total rRNA of cells. The results in Supplementary Fig. S1 show that RNase L activation in experiments of Fig. 1C and G was consistent with OAS3 expression. These results confirm that OAS3, superior to OAS1 and OAS2, could inhibit EV71 replication *in vitro*.

### 3.2 EV71 3C^pro^ reduces expression of OAS3 by proteolytic enzyme activity

**Fig. 2:**
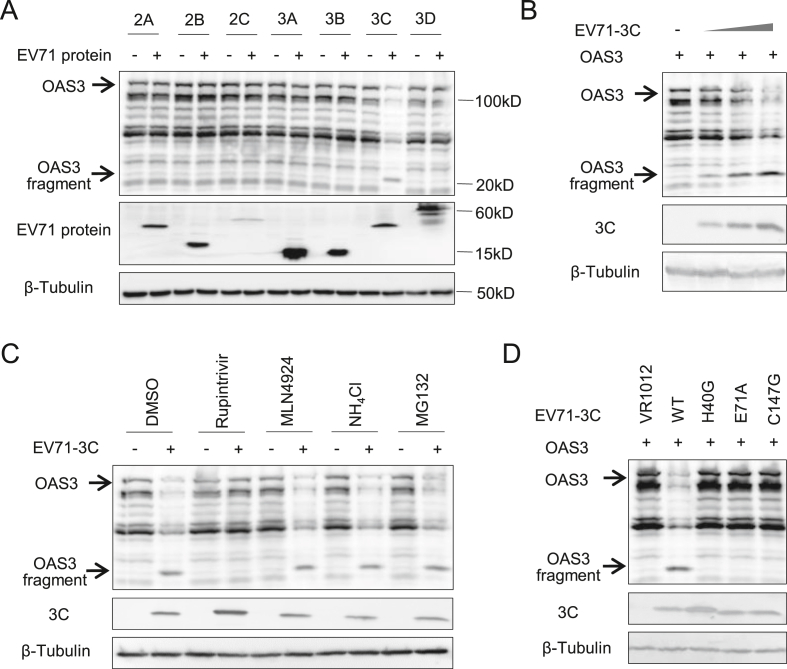
EV71 3C protease reduces expression of OAS3 by proteolytic enzyme activity. **A** HEK293T cells were cotransfected with OAS3 and seven nonstructural protein of EV71 expression plasmids as indicated for 48 ​h prior to harvest. The protein levels of exogenous OAS3 and viral proteins in cell lysates were examined by Western blot. **B** 3C downregulates OAS3 in a dose-dependent manner. HEK293T cells were cotransfected with OAS3 and different amount of EV71-3C expression plasmids (30 ​ng, 100 ​ng, and 300 ​ng) for 48 ​h. The protein levels of exogenous OAS3 and 3C in cell lysates were examined by Western blot. **C** 3C protease inhibitor Rupintrivir restores OAS3 expression. HEK293T cells were cotransfected with OAS3 and EV71-3C expression plasmids as indicated for 18 ​h. Then cells were treated with Rupintrivir, MLN4924, NH_4_Cl, and MG132 for 12 ​h. The protein levels of exogenous OAS3 and 3C in cell lysates were examined by Western blot. **D** The proteolytic activity determines the downregulation effect of EV71-3C on OAS3. HEK293T cells were cotransfected with OAS3 and WT or inactivated mutants of 3C expression plasmids as indicated for 48 ​h prior to harvest. The protein levels of exogenous OAS3 and 3C in cell lysates were examined by Western blot.

Although OAS3 showed definite anti-EV71 activity, we observed that OAS3 could not completely prevent EV71 replication. Moreover, the protein level of intracellular OAS3 was depleted following robust viral replication. The ratio of OAS3/Tubulin gradually decreased from 48 to 72 ​h post-infection (h.p.i) in EV71 infected pLKO.1 ​cells (Fig. 1G). We speculate that EV71 employs an unreported method to reduce protein expression of OAS3. Based on this premise, nonstructural viral proteins of EV71 were co-expressed with OAS3 in HEK293T cells. Compared to other nonstructural proteins, EV71 3C^pro^ efficiently reduced OAS3 expression (Fig. 2A), and in a dose-dependent manner (Fig. 2B). Remarkably, a specific protein fragment (∼20 ​kDa) was observed upon the co-expression of 3C^pro^ but not other EV71 proteins. Inhibitors, including rupintrivir for human rhinovirus 3C^pro^, MLN4924 for NEDD8-activating enzyme, NH_4_Cl for autolysosome, and MG132 for proteasome, were used to verify the pathway of 3C-mediated OAS3 downregulation. We found that rupintrivir, a proven inhibitor of EV71 3C^pro^ (Wang et al., 2011), completely recovered the expression of OAS3 (Fig. 2C). Meanwhile, inactive mutants of 3C^pro^, H40G, E71A, and C147G lost the function of OAS3 downregulation (Fig. 2D). Both rupintrivir and the inactivated mutation of EV 3C^pro^ suppress the appearance of the specific fragments. These results indicated that EV71 3C^pro^ could reduce the protein level of intracellular OAS3 through proteolytic enzyme activity, and might attenuate the antiviral effect of OAS3.

### 3.3 EV71 3C^pro^ cleaves OAS3 at Q982-G983 and enhances virus replication

**Fig. 3:**
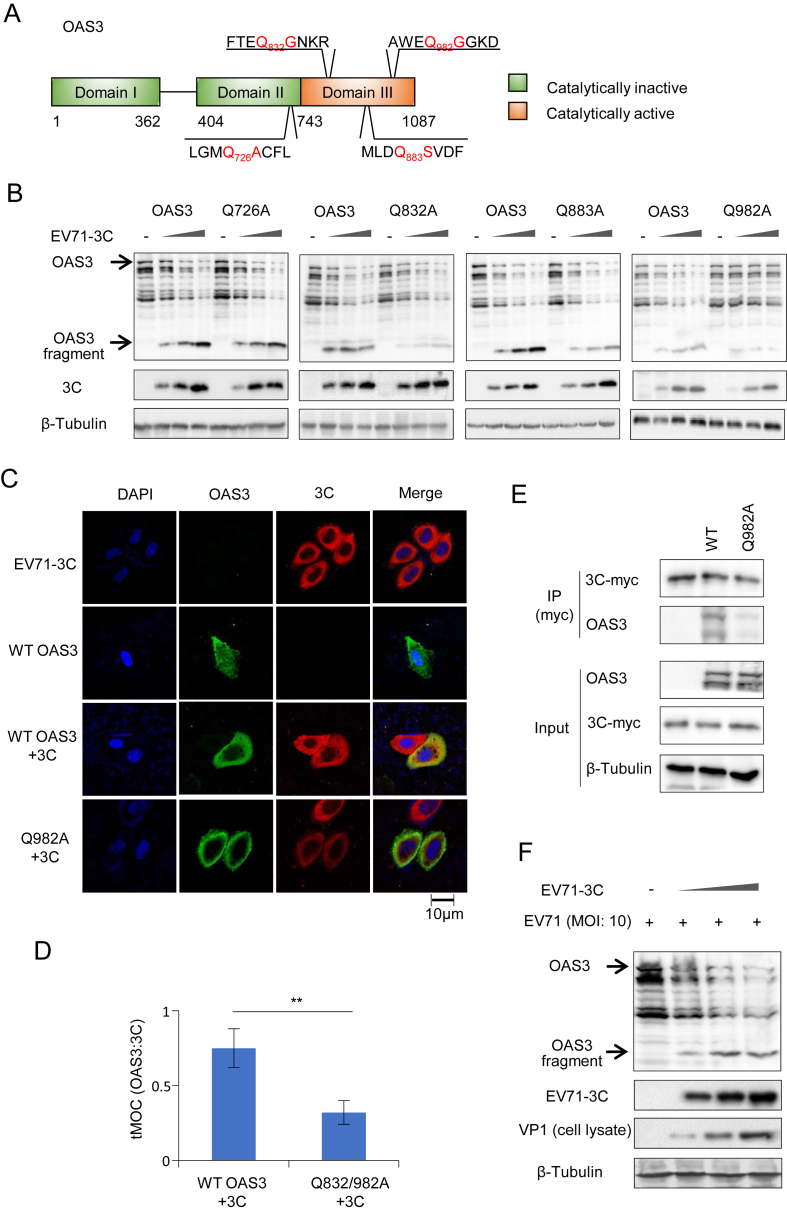
EV71 3C^pro^ cleaves OAS3 at Q982-G983 and enhances virus replication. **A** Amino acid sequences of four possible cleavage sites in OAS3. **B** HEK293T cells were cotransfected with WT OAS3 or OAS3 with four single site mutants and different doses of 3C expression plasmids as indicated for 48 ​h. The protein levels of exogenous OAS3 and 3C in cell lysates were examined by Western blot. **C** EV71 3C^pro^ co-localizes with WT but not Q982A mutant of OAS3. Hela cells were cotransfected with WT or Q982A mutant of OAS3 and 3C expression plasmids as indicated. After 48 ​h of culture, cells were conducted to immunofluorescence based co-localization analysis. Scale bar: 10 ​μm. **D** tMOC as calculated for green signal overlapping with red signal in panel C. **E** Co-IP assay was performed as indicated in HEK293T cells. HEK293T cells were cotransfected with WT OAS3 or Q982A and EV71-3C expression plasmids as indicated for 48 ​h. Cells were harvested and lysed, and then incubated with anti-myc antibody and protein G agarose beads. The cell lysates and eluted proteins were analyzed by Western blot. **F** EV71 3C^pro^ enhanced viral replication. EV71 3C was transfected at different doses into HEK293T cells. Then cells were infected with EV71 at a high MOI of 10 for single round replication. The expression of OAS3 and EV71-VP1 was examined by Western blot.

In general, 3C^pro^ proteolytically cleaves target proteins at Gln/Gly, Gln/Ala, or Gln/Ser scissile pairs (Kitamura et al., 1981). Identifying potential cutting sites is crucial for an in-depth analysis of the relationship between OAS3 and 3C^pro^. Many glutamines within OAS3 resemble the Q-G/Q-S/Q-A target sequence of proteolytic sites for the EV71 3C^pro^. Fortunately, we found that a small C-terminal fragment band of OAS3 (approximately 20 ​kDa with C-terminal HA tag) appeared when OAS3 was co-transfected with EV71 3C^pro^ (Fig. 2). Based on the sizes of the cleaved bands, the Q726-A727, Q832-G833, Q883-S884, and Q982-G983 pairs were identified and tested as potential cleavage sites for 3C^pro^ (Fig. 3A). Four single-point mutants and one double-point mutant of Q to A were constructed. As shown in Fig. 3B, the Q982A mutant of OAS3 showed obvious resistance to the cleavage of 3C^pro^, and the other three mutants showed weak resistance. Meanwhile, immunofluorescence assay showed clear co-localization of EV71 3C^pro^ and wild type (WT) OAS3 in cytoplasm (Fig. 3C). Further calculation of thresholded Mender's overlap coefficient (tMOC) indicated that the mutant Q982A of OAS3 showed lower co-localization with EV71 3C^pro^ compared to the wild type (WT) OAS3 (Fig. 3D). Co-IP indicated that EV71 3C^pro^ interacted with the wild type OAS3 stronger than the mutant Q982A (Fig. 3E). These results show that EV71 3C^pro^ associates with and cleaves OAS3, and the proteolytic motifs Q982-G983 in OAS3 is the key binding and cleavage site of EV71 3C^pro^. When HEK293T cells were transfected with different dosage of EV71 3C^pro^ for 24 ​h, cells were infected with EV71 at a high MOI of 10 for single round replication. As shown in Fig. 3F, the expression of EV71 3C^pro^ reduced the protein level of endogenous OAS3, and enhanced the expression of EV71 VP1 in cell lysate in a dose dependent manner. It indicated that EV71 3C^pro^ could enhance virus replication by down-regulating intracellular OAS3.

### 3.4 OAS3 broadly inhibits the replication of CVB3, CA16, EVD68, and CA6 subtypes

**Fig. 4:**
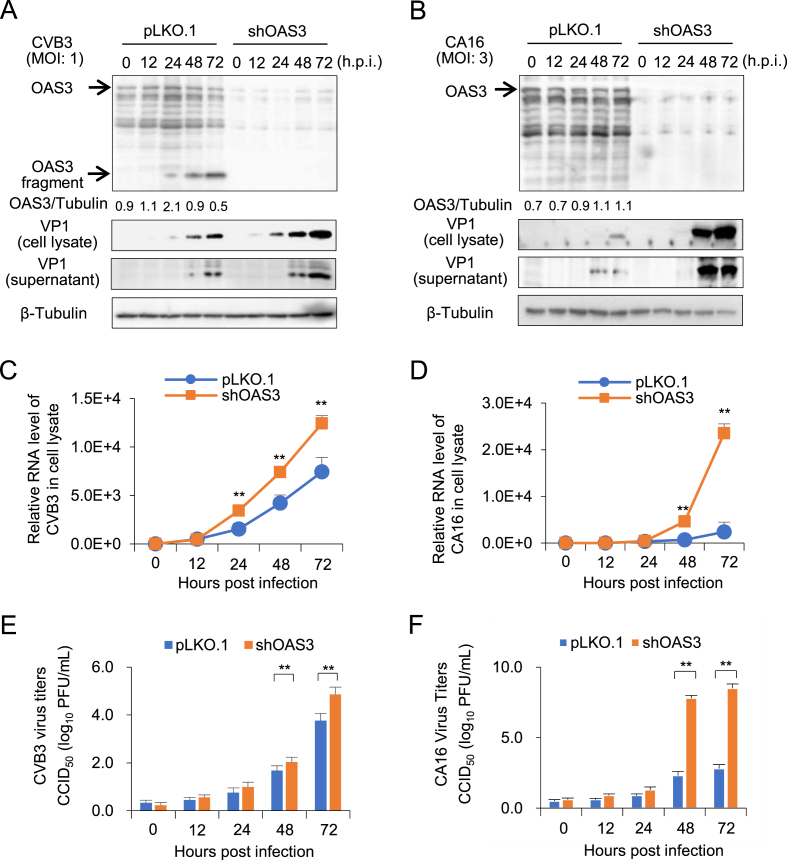
OAS3 suppresses CVB3 and CA16 replication in HEK293T cells. **A** OAS3 knockdown enhances CVB3 replication. shRNA-mediated OAS3 knockdown (shOAS3) and control HEK293T (pLKO.1) cells were infected with CVB3 at an MOI of 1. Cells and supernatants were harvested at the indicated time points. The expression levels of endogenous OAS3 and VP1 were examined by Western blot. **B** OAS3 knockdown enhances CA16 replication. shRNA-mediated OAS3 knockdown (shOAS3) and control HEK293T (pLKO.1) cells were infected with CA16 at an MOI of 3. Cells and supernatants were harvested at the indicated time points. The expression levels of exogenous OAS3 and VP1 were examined by Western blot. **C** CVB3 viral RNA in cell lysate from the experiment of Fig. 4A were examined by RT-qPCR. **D** CA16 viral RNA in cell lysate from the experiment of Fig. 4B were examined by RT-qPCR. **E** CVB3 viral titers in the supernatants from the experiment of Fig. 4A were determined by the plaque assay. **F** CA16 viral titers in the supernatants from the experiment of Fig. 4B were determined by the plaque assay. The results represent the means ​± ​SD from three independent experiments. Statistical significance was analyzed using Student's *t*-test (∗∗*P* ​< ​0.01).

**Fig. 5:**
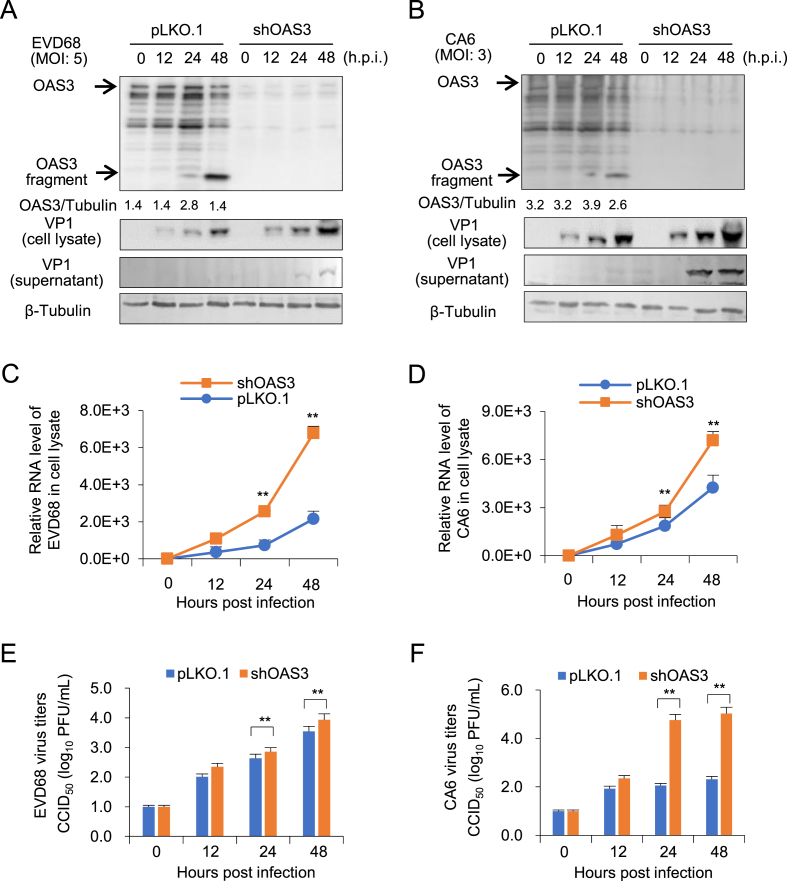
OAS3 suppresses EVD68 and CA6 replication in HEK293T cells. **A** OAS3 knockdown enhances EVD68 replication. shRNA-mediated OAS3 knockdown (shOAS3) and control HEK293T (pLKO.1) cells were infected with EVD68 at an MOI of 5. Cells and supernatants were harvested at the indicated time points. The expression levels of endogenous OAS3 and VP1 were examined by Western blot. **B** OAS3 knockdown enhances CA6 replication. shRNA-mediated OAS3 knockdown (shOAS3) and control HEK293T (pLKO.1) cells were infected with CA6 at an MOI of 3. Cells and supernatants were harvested at the indicated time points. The expression levels of exogenous OAS3 and VP1 were examined by Western blot. **C** EVD68 viral RNA in cell lysate from the experiment of Fig. 5A was examined using RT-qPCR. **D** CA6 viral RNA in cell lysate from the experiment of Fig. 5B was examined using RT-qPCR. **E** EVD68 viral titers in the supernatants from the experiment of Fig. 5A were determined using the plaque assay. **F** CA6 viral titers in the supernatants from the experiment of Fig. 5B were determined using the plaque assay. The results represent the means ​± ​SD from three independent experiments. Statistical significance was analyzed using Student's *t*-test (∗∗*P* ​< ​0.01).

In order to understand whether OAS3 possess the broad anti-EV function, the effect of OAS3 on the replication of enterovirus CVB3, CA16, EVD68, and CA6 subtype was investigated. HEK293T cells transfected with shRNA control (pLKO.1) or shOAS3 were infected by CVB3 (MOI ​= ​1) and CA16 (MOI ​= ​3) for 0–72 ​h, respectively. Western blot and RT-qPCR detection indicated that OAS3 silencing enhanced intracellular viral RNA transcription and protein expression (Fig. 4A–D). Also, extracellular viral titers have been upregulated by OAS3 silencing (Fig. 4E and F). The inhibition effects are consistent with the result of EV71. Similar results were also observed in EVD68 and CA6 infected HEK293T cells (Fig. 5). Taken together, OAS3 can broadly inhibit the replication of multiple EV subtypes such as CVB3, CA16, EVD68, and CA6 *in vitro*.

### 3.5 Key amino acid variations of EV71 3C^pro^ affect its ability to cleave OAS and 3AB

**Fig. 6:**
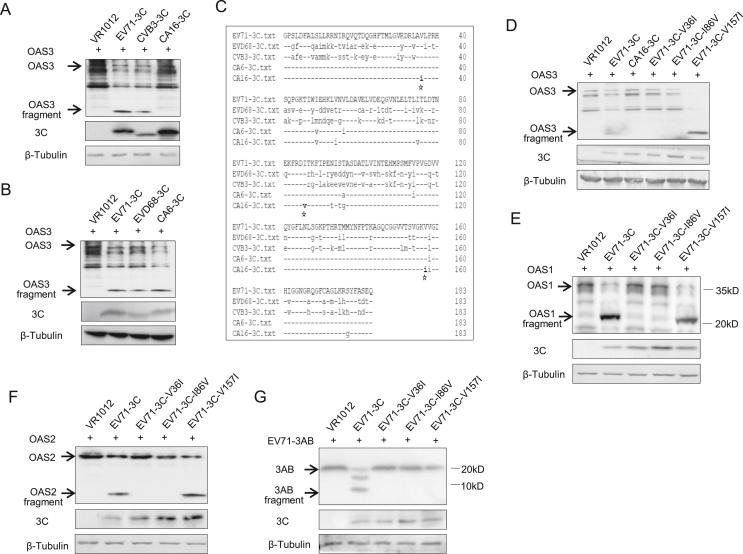
Key amino acid variations of EV71 3C^pro^ affect its ability to cleave OAS and 3AB. **A** HEK293T cells were cotransfected with OAS3 and EV71-3C, CVB3–3C or CA16-3C expression plasmids as indicated for 48 ​h. The protein levels of exogenous OAS3 and 3C in cell lysates were examined by Western blot. **B** HEK293T cells were cotransfected with OAS3 and EV71-3C, EVD68-3C or CA6-3C expression plasmids as indicated for 48 ​h. The protein levels of exogenous OAS3 and 3C in cell lysates were examined by Western blot. **C** Sequence alignment of 3C proteases derived from five representative enterovirus subtypes revealed that amino acids Ile36, Val86, and Ile157 were unique in CA16 3C compared with the other four enterovirus subtypes. **D** HEK293T cells were cotransfected with OAS3 and WT, mutants of EV71-3C, or CA16-3C expression plasmids as indicated for 48 ​h. The protein levels of exogenous OAS3 and 3C in cell lysates were examined by Western blot. **E** HEK293T cells were cotransfected with OAS1 and WT or mutants of EV71-3C expression plasmids as indicated for 48 ​h. The protein levels of exogenous OAS1 and 3C in cell lysates were examined by Western blot. **F** HEK293T cells were cotransfected with OAS2 and WT or mutants of EV71-3C expression plasmids as indicated for 48 ​h. The protein levels of exogenous OAS2 and 3C in cell lysates were examined by Western blot. **G** HEK293T cells were cotransfected with 3AB and WT or mutants of EV71-3C expression plasmids as indicated for 48 ​h. The protein levels of exogenous 3AB and 3C in cell lysates were examined by Western blot.

Although OAS3 has been observed to inhibit the replication of many EVs, CA16 infection does not seem to downregulate the protein level of OAS3 in pLKO.1 ​cells. Correspondingly, the observed expression of VP1 protein of CA16 virus in pLKO.1 ​cells was significantly later than that of the other four viruses (Fig. 4, Fig. 5). Therefore, we hypothesized that 3C^pro^ of different enterovirus subtypes exhibited different OAS3 cleavage ability. And this variation resulted in differences of virus replication in cells. To test this, 3C^pro^ of EV71, CVB3, and CA16 were co-expressed with OAS3 in HEK293T cells. Western blot analysis indicated that CA16 3C^pro^ hardly antagonized OAS3 compared with EV71 or CVB3 3C^pro^ (Fig. 6A). In addition, EVD68 and CA6 3C^pro^ showed cleavage activity similar to that of EV71 3C^pro^ (Fig. 6B). Sequence alignment of 3C^pro^ derived from five representative EV subtypes revealed that three unique amino acids Ile36, Val86, and Ile157, which presented in CA16 3C^pro^, were different from 3C^pro^ from the other four subtypes (Fig. 6C). Mutation of V36I and I86V on EV71 3C^pro^ completely abolished the ability to antagonize OAS3 (Fig. 6D). In addition, the wild type and mutants of EV71 3C^pro^ showed similar function on OAS1 and OAS2 because of the OAS sequence and structural similarity (Fig. 6E and F). We also examined the ability of the wild type and mutants of EV71 3C^pro^ to process the viral protein 3AB, which is important for viral replication. As shown in Fig. 6G, V36I and I86V mutants lost the ability of 3AB processing. Taken together, we suggested that two key amino acid variations Ile36 and Val86 on CA16 3C^pro^ resulted in weak and delayed virus replication of CA16 because of impaired OAS and 3AB cleavage.

## 4 Discussion

The innate immune system is a broad collection of critical intra- and extracellular processes that limit the infectivity of diverse pathogens. The OAS family are important sensors of cytosolic double-stranded RNA that play a critical role in limiting viral infection by activating the latent RNase L to halt viral replication and establish an antiviral state. Among the various viruses examined, *Picornaviridae* show the best correlation between activation of OAS and inhibition of virus replication. For example, Human T98G cells that express OAS1 display resistance to EMC virus replication, but not to VSV replication (Rysiecki et al., 1989; Coccia et al., 1990). Constitutive expression of OAS2 in human HT1080 ​cells causes inhibition of EMC virus replication but not of VSV, SeV, or reovirus replication (Ghosh et al., 2000). Enteroviruses are an important branch of the family of *Picornaviridae*. EV71, as the main pathogen causing hand-foot-mouth disease, is a representative subtype of EV. It is particularly important to study the relationship between EV71 and innate immune system. Previous studies were limited to finding the association between *OAS3* gene polymorphisms and the severity of EV71 infection (Tan et al., 2017; Liu et al., 2018). The effect of OAS3 against EV71 and the mechanism of EV71 escape were not reported. In this study, we firstly investigated the antiviral effect of OAS1, OAS2 and OAS3, and found that OAS3 has a stronger antiviral effect than OAS1 and OAS2 against EV71 (Fig. 1), and exhibits broad spectrum anti-EV activity (Fig. 1, Fig. 4, Fig. 5). This phenomenon is consistent with the fact that OAS3 has better 2-5A synthesis activity than OAS1 and OAS2. Hence, subsequent studies will be needed to demonstrate the role of 2-5A synthesis and RNase L activation in OAS3 against EV71.

Diverse viruses have developed numerous distinct strategies to inhibit upstream or downstream steps of the OAS/RNase L pathway. Some viruses sequester dsRNAs, thereby preventing OAS activation. Examples of proteins with this action include influenza A virus NS1 (Min and Krug, 2006), vaccinia virus (VV) E3L (Chang et al., 1992) and the σ3 outer capsid protein of reoviruses (Huismans and Joklik, 1976), which remarkably plays a structural role in the capsid and also counteracts antiviral responses. The human immunodeficiency virus (HIV) Tat protein binds to tar, a dsRNA structure in the HIV mRNA, to prevent OAS activation by tar (Schröder et al., 1990). Some viruses prevent RNase L activation by degrading 2-5A or production of inactive 2-5A. The Ns2 protein of mouse hepatitis virus (MHV) and VP3 protein of rotavirus prevent RNase L activation to maintain virus replication by degrading 2-5A (Zhao et al., 2012; Zhang et al., 2013). In addition, L∗ protein of Theiler's virus inhibits RNase L activation through direct binding to the enzyme. In the present study, we found that EV71 3C^pro^ could degrade OAS3 by mediating proteolytic cleavage at specific sites (Fig. 2, Fig. 3). Notably, the sequence divergence of EV subtype 3C^pro^ not only determined its cleavage activity for OAS3, but also affected the cleavage of viral protein 3AB. This is the first report that a viral protein directly reduces OAS and escapes the OAS/RNase L system. Although EV71 3C^pro^ has been proven to cleave and process viral precursor protein and host factors, our work illuminated that EV71 3C^pro^ cleaved a novel target OAS3 to enhance viral replication. On the other hand, Han et al. reported the discovery of an RNA structure in the poliovirus (PV) ORF that potently inhibits the endoribonuclease activity of RNase L (Han et al., 2007). ORF 2122 RNA, a fragment of PV 3C ORF, was responsible for the inhibition of RNase L cleavage. The RNA structure associated with the inhibition of RNase L is phylogenetically conserved in group C enteroviruses, including PV1, PV2, PV3, coxsackie A virus 11 (CV11), CV13, CV17, CV20, CV21, and CV24, but is not present in other human enteroviruses (groups A, B, or D enteroviruses). CVB3 mRNA was fully sensitive to cleavage by RNase L. Although no studies have reported whether EV71, CA16, and other enterovirus mRNAs contain the conserved 3C ORF that can resist RNase L cleavage, we believe that EV can escape the antiviral effect of the OAS/RNase L system by means of the unique nucleotide structure and/or proteolytic enzyme activity of the 3C protein. The divergent evolution of EV 3C^pro^ may be relevant to viral escape from the antiviral effect of OAS3. Future studies are necessary to investigate the evolutionary mechanism of 3C^pro^ in viral pathogenesis.

## 5 Conclusions

In this paper, we reveal that OAS3 exhibited broadly anti-viral effect on EV71, CVB3, CA16, EVD68, and CA6. However, EV71, CVB3, EVD68, and CA6, but not CA16 could utilize autologous 3C proteases to cleave intracellular OAS3 and enhance viral replication. Two key amino acids variation Ile36 and Val86 in CA16 3C^pro^ resulted in weak and delayed virus replication of CA16 because of failure of OAS and 3AB cleavage. These findings can be an important entry point for developing novel therapeutic strategies for multiple EVs infection.

## Data availability

All the data generated during the current study are included in the manuscript.

## Ethics statement

This article does not contain any studies with human or animal subjects performed by any of the authors.

## Author contributions

Xiaolei Zhou: conceptualization, formal analysis, investigation, methodology, data curation. Li Tian: formal analysis, investigation, data curation. Jian Wang: investigation and formal analysis. Baisong Zheng: conceptualization, formal Analysis, investigation, writing-original draft, writing-review and editing. Wenyan Zhang: conceptualization, funding acquisition, resources, supervision, writing-review and editing.

## Conflict of interest

The authors declare that they have no conflict of interest.
