# Supplementary figures and images for: EV71 3C protease cleaves host anti-viral factor OAS3 and enhances virus replication
Source: Virol Sin. 2022 May 3;37(3):418–26. doi: 10.1016/j.virs.2022.04.013 (PMC9243667; doi:10.1016/j.virs.2022.04.013)

A

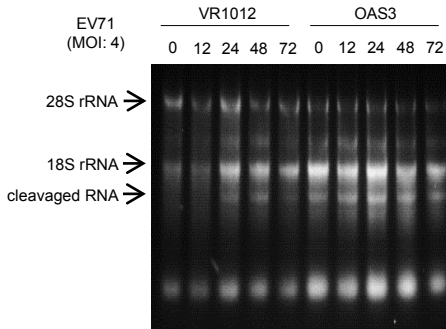

B

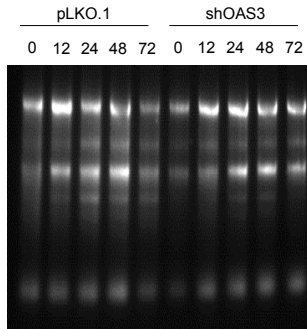

Supplement: Multimedia component 1 [file mmc1.pdf]
