# Supplementary material for: EV71 3C protease cleaves host anti-viral factor OAS3 and enhances virus replication
Source: Virol Sin. 2022 May 3;37(3):418–26. doi: 10.1016/j.virs.2022.04.013 (PMC9243667; doi:10.1016/j.virs.2022.04.013)
Supplement: Multimedia component 2 [file mmc2.docx]

**Virologica Sinica**

**Supplementary Data**

**EV71 3C protease cleaves host anti-viral factor OAS3 and enhances virus replication**

**Xiaolei Zhou^a^, Li Tian^a^, Jian Wang^a^, Baisong Zheng^a^*, and Wenyan Zhang^a^***

*^a^ Center for Infectious diseases and Pathogen biology, Institute of Virology and AIDS Research, Key laboratory of Organ Regeneration and Transplantation of The Ministry of Education, The First Hospital of Jilin University, Jilin, 130021, China.*

*Corresponding authors,

E-mail: zhangwenyan@jlu.edu.cn (W. Zhang), zhengbs@jlu.edu.cn (B. Zheng)

**
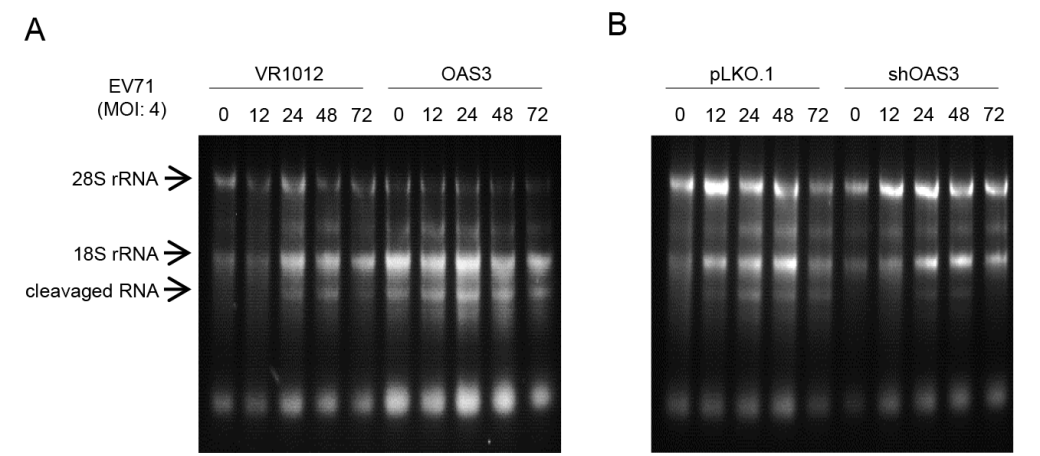
**

**Figure S1**. RNase L activation was examined by agarose electrophoresis of total rRNA of cells. (**A**) The activity of RNase L in the experiment of Figure 1C was measured at corresponding time point upon viral infection. (B) The activity of RNase L in the experiment of Figure 1G was measured at corresponding time point upon viral infection.

Table S1. Primers used for mutation and knockdown in this study.

| Primer name | Primer direction | Sequence (5'-3') |
| --- | --- | --- |
| shOAS3 | Forward | CCGGAAGCCACAAGTCTACTCTACCCTCGAGGGTAGAGTAGACTTGTGGCTTTTTTTG |
|  | Reverse | AATTCAAAAAAAGCCACAAGTCTACTCTACCCTCGAGGGTAGAGTAGACTTGTGGCTT |
| OAS3-Q726K | Forward | GCCCTCGGCATGAAGGCCTGCTTCC |
|  | Reverse | GGAAGCAGGCCTTCATGCCGAGGGC |
| OAS3-Q832A | Forward | AGCCAGTTCACTGAAGCGGGCAATAAAAGGGC |
|  | Reverse | GCTTCAGTGAACTGGCTAAAGCAGCTGAGGAA |
| OAS3-Q883A | Forward | CAGACTATGCTCGATGCGAGCGTAGATTTCG |
|  | Reverse | GCATCGAGCATAGTCTGGCTAGTGAGGCTG |
| OAS3-Q982A | Forward | CCTCACTGTCTATGCATGGGAAGCGGGCGGCAAAGATA |
|  | Reverse | GCTTCCCATGCATAGACAGTGAGGAGTTCGAGGCCGTG |
| EV71 3C-H40G | Forward | CGTACTTCCTCGCGCCAGCCAGCCCGG |
|  | Reverse | GCGCGAGGAAGTACGGCCAGGCGGTCG |
| EV71 3C-E71A | Forward | GGGTGTGAACCTTGCGCTGACCCTGATC |
|  | Reverse | GCAAGGTTCACACCCTGCTCGTCCACC |
| EV71 3C-C147G | Forward | CAAAGCAGGCCAGAGCGGCGGCGTG |
|  | Reverse | TCTGGCCTGCTTTGGTGGGGAAGTTG |

Table S2. Primers used for qRT-PCR in this study

| Primer name | Primer direction | Sequence (5'-3') |
| --- | --- | --- |
| EV71-RT | Forward | CTTTGTGCGCCTGTTTTATAC |
|  | Reverse | GGAAACAGAAGTGCTTGATCA |
| CA16-RT | Forward | CATGCAGCGCTTGTGCTT |
|  | Reverse | CACACAATTCCCCCGTCTTACT |
| CVB3-RT | Forward | GAATGCGGCTAATCCTAACTGC |
|  | Reverse | GCTCTATTAGTCACCGGATGGC |
| Negative-sense-RT | Forward | TTAAAACAGCCTGTGGGTTG |
| Positive-sense-RT | Reverse | Oligo d(T) |
| OAS3-RT | Forward | GAAGGAGTTCGTAGAGAAGGCG |
|  | Reverse | GCATCTCACTGAGGATCTCTGC |
| GAPDH-RT | Forward  Reverse | TGCACCACCAACTGCTTAGC  GGCATGGACTGTGGTCATGAG |
